# Blinding Trachoma: Systematic Review of Rates and Risk Factors for Progressive Disease

**DOI:** 10.1371/journal.pntd.0004859

**Published:** 2016-08-02

**Authors:** Athumani M. Ramadhani, Tamsyn Derrick, Martin J. Holland, Matthew J. Burton

**Affiliations:** 1 London School of Hygiene & Tropical Medicine, London, United Kingdom; 2 Kilimanjaro Christian Medical Centre, Moshi, Tanzania; 3 Moorfields Eye Hospital, London, United Kingdom; RTI International, UNITED REPUBLIC OF TANZANIA

## Abstract

**Background:**

Sight loss from trachoma is the end result of a scarring disease process starting in early childhood and characterised by repeated episodes of conjunctival inflammation (active trachoma). Subsequently, the conjunctiva becomes scarred, causing the eyelashes to turn inwards and scratch the cornea (trichiasis), damaging the corneal surface and leading to corneal opacification and visual impairment. It is thought that this process is initiated and driven by repeated infection with *Chlamydia trachomatis*. We review published longitudinal studies to re-examine the disease process, its progression rates and risk factors.

**Methodology/Principal Findings:**

We searched PubMed for studies presenting incidence and progression data for the different stages of trachoma natural history. We only included studies reporting longitudinal data and identified 11 publications meeting this criterion. The studies were very heterogeneous in design, disease stage, duration, size and location, precluding meta-analysis. Severe conjunctival inflammation was consistently associated with incident and progressive scarring in five studies in which this was examined. One study reported an association between *C*. *trachomatis* infection and incident scarring. No studies have yet demonstrated an association between *C*. *trachomatis* infection and progressive scarring. Several studies conducted in regions with low prevalence active disease and *C*. *trachomatis* infection found evidence of on-going scarring progression.

**Conclusions/Significance:**

Overall, there are few longitudinal studies that provide estimates of progression rates and risk factors, reflecting the challenges of conducting such studies. Our understanding of this disease process and the long-term impact of control measures is partial. Intense conjunctival inflammation was consistently associated with scarring, however, direct evidence demonstrating an association between *C*. *trachomatis* and progression is limited. This suggests that on-going chlamydial reinfection may not be mandatory for progression of established scarring, indicating that sight threatening trichiasis may continue to evolve in older people in formerly endemic populations, that will require service provision for years after active disease is controlled.

## Introduction

**Fig 1:**
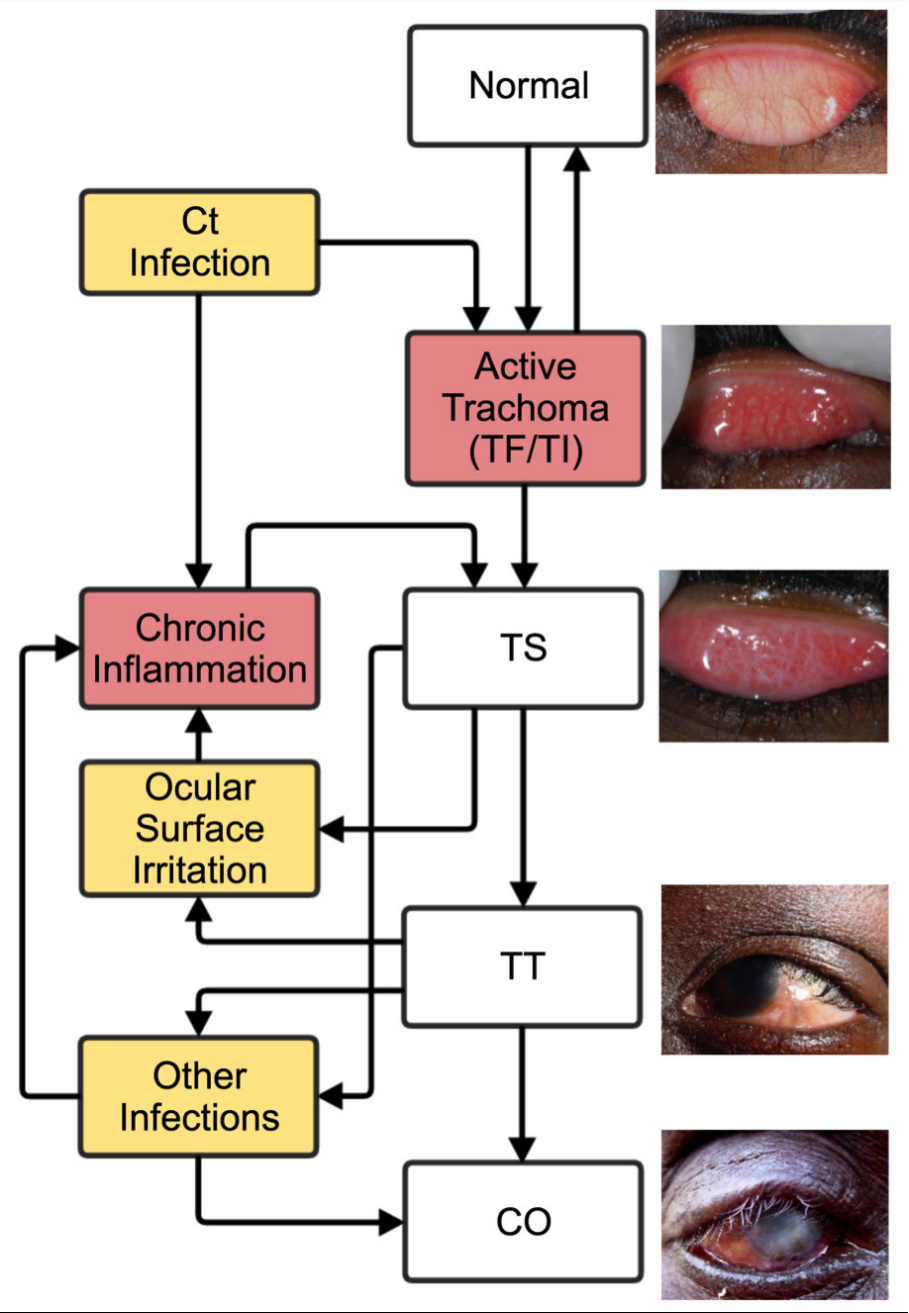
Natural history of trachoma: Normal healthy tarsal conjunctiva without inflammation, follicular trachoma (TF), intense inflammatory trachoma (TI), scarring trachoma (TS), trichiasis (TT) and corneal opacification (CO). Ct: *Chlamydia trachomatis*.

Sight loss from trachoma is the end result of a scarring disease process. The widely accepted view is that it follows the natural history illustrated in Fig 1 [1]. Trachoma is caused by the obligate intracellular bacterium *Chlamydia trachomatis*. In a typical endemic setting, repeated chlamydial infection of the conjunctiva starts early in life. This can initiate recurrent episodes of chronic conjunctival inflammation, characterised by the formation of lymphoid follicles most easily seen in the upper tarsal conjunctival surface. In the Simplified WHO grading system this is referred to as Trachoma Inflammation—Follicular (TF), which is equivalent to F2 and F3 of the detailed WHO-FPC grading system [2, 3]. In some cases the inflammation can be more marked, with severe papillary hypertrophy, referred to as Trachoma Inflammation—Intense (TI) in the simplified system and P3 in the detailed system [2, 3]. These signs of “Active Trachoma”, TF and TI, are most frequently found in younger children, becoming less prevalent with increasing age. They are characterised by a cell-mediated adaptive immune response to *C*. *trachomatis* [4]. With time trachomatous conjunctival scarring (TS) gradually develops as a result of inflammation-induced tissue damage [2, 4]. The Simplified WHO grading of TS is taken to be equivalent to the grades C1-C3 of the Detailed WHO grading system [2, 3]. In highly endemic regions TS can develop in childhood, whereas in less endemic regions it usually becomes visible from early adulthood.

Chronic conjunctival inflammation is also frequently found in adults with established conjunctival scarring, Fig 1. This later stage conjunctival inflammation is usually not follicular in nature, *C*. *trachomatis* infection is rarely detected and it is characterised by a prominent innate epithelial immune response [4–6]. It is possible that non-chlamydial infections, dryness and irritation of the scarred conjunctiva also contribute to driving inflammation and progression in the context of established scarring, Fig 1 [4].

Eventually the conjunctival scarring causes the eyelashes to turn in, such that they now scratch the surface of the eye, which is referred to as trachomatous trichiasis (TT) [2]. If the trichiasis is left uncorrected it traumatises the surface of the cornea resulting in sight loss from corneal opacification (CO). Again other pathogens (bacteria and fungi) may contribute to the development of corneal scarring through secondary infections, Fig 1.

The most recent World Health Organization (WHO) estimates suggest that about 232 million people live in trachoma endemic areas in 51 countries [7]. Approximately 1.2 million people are irreversibly blind from trachoma. A further 40 million are thought to be affected by active trachoma, and are at risk of developing scarring and blindness [8]. To meet this large public health challenge, the WHO-led Global Alliance for the Elimination of Trachoma by 2020 recommends the implementation of the SAFE strategy which tackles the disease at different stages: Surgery to correct trichiasis, Antibiotics to treat chlamydial infection and Facial cleanliness and Environmental improvements to suppress transmission of infection [1].

Despite many years of research, there are relatively few detailed longitudinal studies that investigate the rates and determinants of disease progression through these stages. An understanding of this process is relevant to disease control measures and planning. In this review we draw together and re-examine the literature on the natural history of trachoma, to identify the rates and risk factors for progression through the stages of this disease process.

## Methods

References were identified through searches of PubMed for articles published at any date, by use of the terms (i) “trachoma” AND (ii) “scarring”, “trichiasis”, “cornea opacity”, “*Chlamydia trachomatis*”, “visual impairment” OR “blindness”. Articles resulting from these searches and relevant publications cited in these articles were reviewed. We included longitudinal studies which: (1) reported the incidence and/or progression of conjunctival scarring; (2) the incidence and/or progression of trachomatous trichiasis; (3) the incidence and/or progression of corneal opacification; (4) the incidence and/or progression of visual impairment and blindness from trachoma. For the purposes of this review, we define incident disease (conjunctival scarring, trichiasis or corneal opacification) to be the development of these signs in individuals who did not previously have evidence of them. We reviewed the bibliographies of publications meeting these inclusion criteria for any additional publications that might also meet them. This search yielded 1595 articles and one author screened the titles and abstracts of these for potentially eligible publications. Two authors (AR and MB) jointly reviewed potential articles for eligibility and extracted the data.

For each disease progression stage we extracted and recorded core information using a standardised form and present this in the Tables. The core information included country, year of publication, population active disease level, study design, study population, duration of follow-up, numbers of participants, number and proportion progressing, grading system, use of antibiotic for infection control. Additional information on specific associations with disease progression and clinical inflammation, *C*. *trachomatis* infection, gender and age were collected if reported. For the purpose of this review the prevalence levels of TF in the populations or regions studied were categorised as follows: Hypoendemic <10%, Mesoendemic 10–20% and Hyperendemic >20% [9]. For some reports data on active disease prevalence was not presented, therefore, we have categorised the active disease prevalence level for such studies based on other contemporary sources of information about disease prevalence for the same regions. Clinical signs are reported using the specific trachoma grading systems used in the study (details provided in Tables 1–5), these were usually either the Simplified or the Detailed WHO Trachoma Grading Systems [2, 3]. Due to the heterogeneity of the study designs and reports a meta-analysis was not performed.

**Table 1:** Progression from active to scarring trachoma.

Country / Year	Study design	Participants	Progression to TS	Comments
Tunisia, 1990 [10]	Prospective 14-year study of the resident population of a trachoma-endemic Tunisian village. Conducted to identify clinical signs and environmental factors associated with development of scarring. A random sample of people seen at baseline were re-examined at 14-years.	Baseline, 1969–72: 2000 people, of all ages. Follow-up, 1986–87: 213 people who were aged 1 month to 32 years at baseline. Loss to follow-up: Information not provided. Baseline Scarring: • C0 82 (38.7%) • C1 51 (24.0%) • C2 58 (27.4%) • C3 21 (9.9%)	• Baseline: 82 had no scarring (C0). • At 14 years 14/82 (17.1%) had developed severe scarring (C3) • Incident TS rate: 1.2%/year	• Hyperendemic setting (Regional survey data) • No previous MDA, however during the 1980’s systematic antibiotic treatment was carried out which dramatically reduced active trachoma.[35] • Clinical grading: detailed WHO-FPC system.[3] • The data presentation and analysis in this report are relatively limited. However, it represents the first long term study of the relationship between inflammation and subsequent scarring. The report only includes the rate of transition from no scarring (C0) to C3 (the most severe grade with distortion), it is likely that many other individuals without scarring at baseline would have gone on to develop some lesser degree of scarring (C1 or C2). Data not disaggregated by gender. • Predictors of severe scarring (C3) at 14 years (whole group of 213): ○ Baseline TF (F2/F3): RR 2.8 ○ Baseline TI (P3): RR 18 ○ Household density (closeness of houses): RR 1.3
Tanzania, 1997 [14]	Mathematical model of the 5-year incidence of TS in women. Using age-stratified cross-sectional data on the different clinical stages.	• 4898 women in survey. • Loss to follow-up: N/A	• Incident TS (model) by age group: 15–19 years: 3.1% / 5 yrs; 55–59 age: 14.3% / 5 yrs • Incident TS rate: 15–19 years: 0.6%/year; 55–59 years: 4.9%/year	• Hyperendemic setting • No previous MDA. • Clinical grading: simplified WHO system.[2]
Tanzania, 2001 [11]	Prospective cohort study of “constant severe trachoma” in the development of incident trachoma scarring in children. Cases of “constant severe trachoma” were defined by the presence of severe inflammatory trachoma (TI) on at least 3 out of 4 examinations during the baseline year. The comparison group was defined as children “without constant severe trachoma” (up to 2 episodes of TI out of 4). The two groups were matched by age, gender and neighbourhood. Swabs were collected for *C*. *trachomatis* infection by PCR at 7 years only.	• Baseline, 1989: Age 1–7 years; Cases 118; Comparison 118. • Follow-up, 1996: Age 8–14 years; Cases 96; Comparison 94 • Loss to follow-up: 42	• 7 year Incident TS: Cases: 28/96 (29.2%); Comparison 9/94 (9.6%). • Incident TS rate: Cases: 4.2%/year; Comparison: 1.4%/year	• Hyperendemic setting. (Regional survey data) • No previous MDA. After baseline MDA with topical tetracycline eye ointment was administered for 30 days.[36] • Clinical grading: simplified WHO system.[2] • Predictors of incident scarring at 7 years: ○ Age (per year): OR 1.32, 95% CI 1.07–1.62 ○ Female: OR 2.49, 95% CI 1.02–6.08 ○ Constant severe trachoma: OR 4.85, 95% CI 2.05–11.4 • Incident scarring was highest in those who had TI and lowest in those that had TF at the 7-year follow-up (1996): ○ Cases: TF 1/30 (3.3%), TI 22/40 (55.0%), None 5/26 (19.2%) ○ Comparison: TF 1/30 (3.3%), TI 5/13 (38.5%), None 3/51 (5.9%) • *C*. *trachomatis* infection at the 7-year time point was more frequently detected in individuals that developed scarring: TS 12/26 (42.9%) vs no TS 20/67 (29.9%), OR 2.48, p = 0.02. The clinical / infection status of individuals from the two groups was unknown during the 7 year interval.
Tanzania, 2009 [12]	Prospective cohort study of the impact of *C*. *trachomatis* infection and severe trachoma on development of scarring. Participants were all children under 10 years (at baseline) living in a single village; examined at baseline, 2, 6, 12, 18 months and 5 years. Definitions: • “Constant infection”: infection on at least 3 of 5 visits during the initial 18 months. • “Constant severe trachoma”: severe trachoma (10+ follicles or TI or both) on at least 3 of 5 visits during the initial 18 months. • “Constant infection and constant severe trachoma”: combination of the above.	• Cohort of 189 children, aged 0–9 years at baseline (2000). • Loss to follow-up: 6	• Baseline TS prevalence: 6/189 (3.0%) • Incident TS by 5 years: 32/183 (17.4%) • Incident TS rate: 3.5%/year	• Hyperendemic setting (70% TF at baseline) • Clinical grading: simplified WHO system with additional more detailed grading of scarring on photographs.[2, 13] • MDA was administered at baseline and at 18 months. • Chlamydia test: Amplicor PCR, Roche • Baseline: ○ Scarring: male; 2/90 (2.1%), female; 4/99 (4.0%) ○ *C*. *trachomatis* infection: male; 35/90 (38.8%), female 43/99 (43.4%) • Incident TS at 5 years by disease/infection group: ○ No infection/disease: 4/59 (6.8%) ○ Sporadic infection/disease: 12/79 (15.2%) ○ Constant severe trachoma only: 7/20 (35.0%) ○ Constant infection only: 4/9 (44.4%) ○ Constant severe trachoma and constant infection: 5/16 (31.2%) • Significant predictors of incident scarring at 5 years (multivariable logistic regression model): ○ Age (per year) OR 1.26, 95% CI 1.08–1.47 ○ Gender (female) OR 2.55, 95% CI 1.13–5.75 ○ Sporadic infection/disease (relative to no infection/disease): OR 1.76, 95% CI 0.48–6.50 ○ Constant infection and/or severe disease (relative to no infection/disease): OR 5.74, 95% CI 2.39–13.77
Tanzania, 2009 [13]	Prospective cohort study of incident and progressive scarring. Participants were individuals of all ages, examined at baseline and 5-years.	• Baseline (2000): 990 people of all ages. • Follow-up (2005): 487 people of which 453 had gradable images from all time points. • Loss to follow-up: 437	• Baseline TS prevalence: 86/453 (18.9%) • Incident TS by 5 years: 75/367 (20.4%) • Incident TS rate: 4.1%/year	•Hyperendemic setting (70% TF at baseline) • Clinical grading: simplified WHO system with additional more detailed grading of scarring on photographs.[2, 13] • MDA was administered at baseline and at 18 months. • There was a trend of increasing incidence with age, p = 0.038

**Table 2:** Progression of scarring trachoma.

Country / Year	Study design	Participants	Progression of TS to TS+	Comments
Tunisia, 1990 [10]	Prospective 14-year study of the resident population of a trachoma-endemic Tunisian village. Conducted to identify clinical signs and environmental factors associated with development of scarring. A random sample of people seen at baseline were re-examined at 14-years.	Baseline, 1969–72: 2000 people of all ages. Follow-up, 1986–87: 213 people who were aged 1 month to 32 years at baseline. Loss to follow-up: Information not provided. Baseline Scarring: • C0 82 (38.7%) • C1 51 (24.0%) • C2 58 (27.4%) • C3 21 (9.9%)	Progressive Scarring, by 14 years: “worse scarring” reported in 146/213 (68.5%): • C0 to C3: 14/82 (17.1%) • C1 to C3: 10/51 (19.2%) • C2 to C3: 40/58 (69.0%) Progressive TS rate: • C0 to C3: 1.2%/year • C1 to C3: 1.4%/year • C2 to C3: 4.9%/year	• Hyperendemic setting (Regional survey data) • No previous MDA, however during the 1980’s systematic antibiotic treatment was carried out which dramatically reduced active trachoma.[35] • Clinical grading: detailed WHO-FPC system.[3] • The data presentation and analysis in this report are relatively limited. The “worse scarring” analysis appears to include incident scarring cases as well as deterioration of established scarring. Not possible to sub-divide the presented data. Data not disaggregated by gender. • Predictors of severe scarring (C3) at 14 years (whole group of 213): ○ Baseline TF (F2/F3): RR 2.8 ○ Baseline TI (P3): RR 18 ○ Household density (closeness of houses): RR 1.3
Tanzania, 2009 [13]	Prospective cohort study of incident and progressive scarring. Participants were people of all ages, examined at baseline and 5-years.	• Baseline, 2000: 990 people of all ages. • Follow-up, 2005: 487 people of which 453 had gradable images from all time points. • Loss to follow-up: 437	• Baseline TS prevalence: 86/453 (18.9%). • Progressive TS by 5 years: 40/85 (47.1%) • Progressive TS rate: 9.4%/year	• Hyperendemic setting (70% TF at baseline) • Clinical grading: simplified WHO system with additional more detailed grading of scarring on photographs.[2] • MDA was administered at baseline and at 18 months. • There was no evidence for a difference in the proportion showing progression with age
Ethiopia, 2015 [17]	Prospective cohort study of progressive scarring in adults with established scarring and minor trichiasis (<6 lashes touching the eye). Examined and swabs collected every 6 months for two years. Swabs were analysed for *C*. *trachomatis* infection and expression of several genes potentially involved with inflammation and scarring. Progressive scarring was determined by direct comparison of baseline and two year photographs.	• Baseline, 2008: 650 participants. • 585 people had paired photographs from baseline and 24 months. • Loss to follow-up: 65	• Progressive scarring by 2 years: • 135/585 (23.1%) • Progressive TS rate: 11.6%/year.	• Hyperendemic setting (Regional survey data) • Clinical grading: detailed WHO-FPC system, with more detailed grading of scarring on photographs.[3] • MDA had been delivered in this region of Ethiopia several times before the start of the study and during the two year period. • Progressive scarring was strongly associated with and increasing number of inflammatory (P2/P3) episodes: OR 5.93, 95%CI 3.3–10.6, p<0.0001. • There was no association between scarring progression and age, gender or body mass index. No episode of *C*. *trachomatis* infection were detected. • Gene expression analysis (106 progressors vs 106 non-progressors): clinical inflammation (not scarring progression) was associated with increased expression of *S100A7*, *IL1B*, *IL17A*, *CXCL5*, *CTGF*, *CEACAM5*, *MMP7*, *CD83* and reduced *SPARCL1*.
Tanzania, 2015 [17]	Prospective cohort study of progressive scarring in adults with established scarring. Examined and swabs collected every 6 months for two years. Swabs were analysed for *C*. *trachomatis* infection and expression of several genes potentially involved with inflammation and scarring. Progressive scarring was determined by direct comparison of baseline and two year photographs.	• Baseline, 2009: 804 participants • 577 people had paired photographs from baseline and 24 months	• Progressive scarring by 2 years: • 173/577 (30.0%) • Progressive TS rate: 15.0%/year.	• Hypoendemic setting (Regional survey data) • Clinical grading: detailed WHO-FPC system, with more detailed grading of scarring on photographs.[3] • No MDA has been delivered to this region. • Progressive scarring was strongly associated with and increasing number of inflammatory (P2/P3) episodes: OR 5.76, 95% CI 2.6–12.7, p<0.0001. • No association between scarring progression and gender or body mass index. • Progressors were a bit older: 50.9 years vs 43.8 years, p<0.0001. *C*. *trachomatis* infection was very rare and not associated with progression. • Gene expression analysis (97 progressors vs 97 non-progressors): clinical inflammation (not progressive scarring) was associated with increased expression of *S100A7*, *IL17A*, *CXCL5*, *MMP7 and CEACAM5*. Only *IL1B* (Fold Change 1.54, p = 0.0067) and *S100A7* (Fold Change 1.43, p = 0.027) had associations with progressive scarring of marginal significance.

**Table 3:** Progression to trachomatous trichiasis.

Country / Year	Study design	Participants	Progression of TS to TT	Comments
Tunisia, 1990 [10]	Prospective 14-year study of the resident population of a trachoma-endemic Tunisian village. Conducted to identify clinical signs and environmental factors associated with development of scarring. A random sample of people seen at baseline were re-examined at 14-years.	Baseline, 1969–72: 2000 people of all ages. Follow-up, 1986–87: 213 people who were aged 1 month to 32 years at baseline. Loss to follow-up: Information not provided. Baseline Scarring: • C0 82 (38.7%) • C1 51 (24.0%) • C2 58 (27.4%) • C3 21 (9.9%)	Progression to TT occurred in 17/213 (8.0%). The risk of progression to TT was related to the baseline scarring severity: • C0 1/82 (1.2%) • C1 0/51 (0%) • C2 8/58 (13.8%) • C3 8/21 (38.1%) Progression from TS to TT: 0.6%/year • C0: 0.1%/year • C1: 0.0%/year • C2: 1.1%/year • C3: 2.7%/year	• Hyperendemic setting (Regional survey data) • No previous MDA, however 1980s systematic antibiotic treatment was carried out which dramatically reduced active trachoma[35] • Clinical grading: detailed WHO-FPC system.[3] • The data presentation and analysis in this report are relatively limited. The risk of developing TT was greater with increasing baseline scarring and inflammation.
Tanzania, 1997 [14]	Mathematical model of the 5 year incidence of TT in women. Using age-stratified cross-sectional data on the different clinical stages.	• 4898 women in survey. • Loss to follow-up: N/A	• Incidence of TT by age in all women: 15–19 age 0.3% / 5 years 55–59 age 6.4% / 5 years • Incidence of TT by age in women with TS at baseline: 15–19 age 3.2% / 5 years; 55–59 age 15.1% / 5 years • Incident TT rate in all women: 15–19 years: 0.06%/year; 55–59 years: 1.3%/year. • Incident TT rate in women with TS: 15–19 years: 0.6%/year; 55–59 years: 3.0%/year	• Hyperendemic setting • No previous MDA. • Clinical grading: simplified WHO system.[2]
Tanzania, 1999 [19]	Prospective 7 year cohort study to measure the incidence of TT in women with and without baseline conjunctival scarring. The cohort was recruited from six villages. All women were examined for TS and TT at baseline (1989). Seven years later all available women who had TS (without TT) at baseline and a similar sized random sample of those without any TS were re-examined.	• Cohort participants who completed the follow-up at 7 years: 523 with TS at baseline. 503 without TS at baseline. • Loss to follow-up: 468	• Incidence of trichiasis at 7-years by baseline TS status: TS: 9.2% / 7 years No TS: 0.6% / 7 years. • Incident TT rate: 1.3%/year in women with pre-existing scarring. 0.1%/year with no TS	• Hyperendemic setting (Regional survey data) • No previous MDA • Clinical grading: simplified WHO system.[2] • Predictors of trichiasis: ○ Age (increase per year): OR 1.03, 95%CI: 1.01–1.06 ○ Infection at follow up: OR 2.51, 95%CI: 1.1–5.69
Gambia, 2001 [20]	The 1986 Gambian National Blindness and Eye Disease Survey was a 1% sample of the total population. In this 8174 people were examined. 12 years later individuals who were found to have TS in 1986 were retraced and assessed for the development of TT and CO.	• Baseline, 1986: 639/8174 people ≥18 years were found to have TS • Follow-up, 1998: 326/639 (51%) were re-examined • Loss to follow-up: 313	• Progressed from scarring to trichiasis: 19/297, 6.4% / 12 years. • Incident TT rate in people with TS: 0.5%/year	• Hypoendemic setting (Regional survey data) • No previous MDA • Clinical grading: simplified WHO system.[2] • Risk factor for trichiasis: ○ Old age: OR 1.07, 95% CI 1.01–1.12
Gambia, 2010 [21]	Five year prospective study of the population of 14 adjacent Gambian villages.	• Baseline, 2001: 592 people >15 years • 5-years, 2006: 456 people >15 years • Loss to follow-up: 136	• Baseline TT: 9/592 (1.5%). • 5-year TT: 6/456 (1.3%) • Incident TT Cases: 2/456, 0.4% / 5 years • Incident TT rate in all adults: 0.1%/year	• Mesoendemic setting (15% TF at baseline) • No previous MDA • Clinical grading: detailed WHO-FPC system.[3] • At 5-years, 3/6 case had TT at baseline and 1/6 was a new resident

**Table 4:** Progression of trachomatous trichiasis.

Country / Year	Study design	Participants	Progression of TT to TT+	Comments
Gambia, 2002 [22]	One year longitudinal study of individuals with TT in at least one eye.	• Baseline, 1996: 190 people. • Major trichiasis 135; Minor trichiasis 55. • Follow-up, 1997: 169 people were re-examined at 12 months. • Loss to follow-up: 21	• Progression of Minor to Major trichiasis: 18/55 (33%) / 1 year • Progression of unilateral to bilateral TT: 21/46 (46%) / 1 year	• Hypoendemic setting (Regional survey data) • No previous MDA. • Clinical grading: simplified WHO system.[2]
Gambia, 2006 [23]	Four year longitudinal study of individuals with TT in at least one eye, who had declined surgery. Examined at baseline and 4 years.	• Baseline, 1996: 220 people • Follow-up, 2000: 153 people were re-examined • Loss to follow-up: 67	• Progression of Minor to Major trichiasis: 28/75 (37.3%) eyes. • Progression Rate: 9.3%/year • Progression of unilateral to bilateral trichiasis: 12/42 (29%) eyes • Progression rate: 7.3%/yr	• Hypoendemic setting (Regional survey data) • No previous MDA • Clinical grading: detailed WHO-FPC system.[3] • Univariate association between TT progression and conjunctival inflammation (P2 or P3) at 4-years: OR 3.07, 95%CI 1.23–7.70, p *=* 0.017. This was not significant in a logistic regression model. • *C*. *trachomatis* was detected in 2/146 (1.4%) tested at 4-years and was not associated with TT progression.
Ethiopia 2011 [24, 25]	Two year prospective randomised controlled trial of epilation vs surgery for Minor trichiasis (<6 lashes). 650 individuals were randomised to the epilation arm at baseline and followed every six months for two years. Outcome measure was presence of 5+ lashes. At two years all were offered TT surgery, 383 chose to continue epilating and were followed up for an additional two years.	• Baseline, 2008: 650 people • Primary outcome data available for 637 people at 2 years (2010). • Follow-up, 2012: 383 people who continued epilating were re-examined. Loss to follow-up: at 2 years: 13; at 4 years: 267	• At 2 years: progression of Minor to Major trichiasis: 84/637 (13.2%) eyes. • Progression rate: 6.6% / year. • At 4 years, comparing baseline to four years, 82 /383 (21.4%) had more eyelashes touching and 200/383 (52.2%) had fewer.	• Hyperendemic setting (Regional survey data) • Clinical grading: detailed WHO-FPC system, with more detailed grading of scarring on photographs.[3] • MDA had been delivered in this region of Ethiopia several times before the start of the study and during the two year period.

**Table 5:** Progression to corneal opacification, visual impairment and blindness.

Country / Year	Study design	Participants	Progression of TT to CO	Comments
Tanzania, 1997 [14]	Mathematical model of the 10 year incidence of CO in women. Using age-stratified cross-sectional data on the different clinical stages.	• 4898 women in survey. • Loss to follow-up: N/A	Incidence of CO, attributable to trachoma: All women: • 15–24 yrs 0.16% / 10 years • 45–54 yrs 2.80% / 10 years Progression rate: • 15–24 yrs 0.02%/year • 45–54 yrs 0.3%/year Women with TT: • 15–24 yrs 27.2% / 10 years • 45–54 yrs 53.5% / 10 years Progression rate: • 15–24 yrs 2.72%/year • 45–54 yrs 5.35%/year	• Hyperendemic setting • No previous MDA • Clinical grading: simplified WHO system.[2] • In this model around half of all corneal opacity was due to causes other than trachoma. In women under 35 years these other causes dominated. In older ages trachoma was the main cause.
Gambia, 2001 [20]	The 1986 Gambian National Blindness and Eye Disease Survey was a 1% sample of the total population. In this 8174 people were examined. 12 years later the people who were found to have TS in 1986 were retraced to assess them for the development of TT and CO.	• Baseline, 1986: 639/8174 people ≥18 years were found to have TS • Follow-up, 1998: 326/639 (51%) were re-examined • Loss to follow-up: 313	• Progressed from TS to CO: 18/302 (5.9%) / 12 years. • Progression rate: 0.5%/year • Progressed from TT to CO: 4/20 (20%) / 12 years. • Progression rate: 1.7%/year	• Hypoendemic setting (Regional survey data) • No previous MDA • Clinical grading: simplified WHO system.[2] • Risk factor for corneal opacity: ○ Trichiasis at baseline: OR 8.4, 95% CI 1.8–39.2 • Old age: OR 1.07, 95% CI 1.01–1.12 • Progressed from TS to incident visual impairment / blindness: ○ 53/321 (16.5%), all causes ○ 8/321 (2.5%), attributed to cornea scarring • Progressed from TT to incident visual impairment / blindness: ○ 4/26 (15.4%), all causes ○ 2/26 (7.7%), attributed to cornea scarring
Gambia, 2002 [22]	One year longitudinal study of individuals with TT in at least one eye. Progression was considered significant if the baseline cornea grading had been CC0 / CC1 and the 1-year grade was CC2 / CC3.	• Baseline, 1996: 190 people. Major TT 135. Minor TT 55. • Follow-up, 1997: 169 people were re-examined at 12 months • Loss to follow-up: 21	• Incident CO in individuals with TT: 10/104 (10%) / 1 year • Progressive CO in individuals with un-operated Major TT: 33/96 (34%) / 1 year	• Hypoendemic setting (Regional survey data) • No previous MDA • Clinical grading: detailed WHO-FPC system used for cornea grading.[3] • Change in vision over one year: ○ 8/88 (9%) had incident visual impairment or blindness. • There was a non-significant trend to more visual deterioration with major TT (9%) compared to minor TT (4%) at baseline.
Gambia, 2006 [23]	Four year longitudinal study of individuals with TT in at least one eye, who had declined surgery. Examined at baseline and 4 years.	• Baseline, 1996: 220 people • Follow-up, 2000: 153 people were re-examined, with 241 eyes that had not been surgically treated. • Loss to follow-up: 67	• Incident CO in eyes with TT at baseline: 16/211 (7.6%) / 4 years. • Progression rate: 1.9%/year	• Hypoendemic setting (Regional survey data) • No previous MDA • Clinical grading: detailed WHO-FPC system.[3] • At baseline 30/241 eyes had CO. • CO was only found in eyes with TT. New CO was associated with the presence of Major TT at 4-years (14/16 had major TT). Incident CO by 4 years was more frequent in eyes that had Major TT at baseline: ○ Minor or no TT at baseline: 6/117 (5.1%) ○ Major TT at baseline: 10/99 (10.1%). • There was an overall deterioration in visual acuity over the 4 years, of 0.22 LogMAR unit. This change was more marked (non-significant) for eyes with TT (0.30) than for those without TT (0.15). 29/221 eyes had newly deteriorated to <3/60. However, only 6/29 were due to CO, the large majority were due to cataract.
Ethiopia, 2011 [17]	Two year prospective randomised controlled trial of epilation vs surgery for Minor trichiasis (<6 lashes). 650 individuals were randomised to the epilation arm at baseline and followed every six months for two years. The change in CO was assessed by direct comparison on digital photographs.	• Baseline, 2008: 650 people • Primary outcome data available for 637 people. • Loss to follow-up: 13	• Change in CO, in people with Minor trichiasis who were epilating at 2 years: • Increased CO: 33/603 (5.5%) / 2 years; 2.75%/year • Reduced CO: 7/603 (1.2%) / 2 years; 0.6%/year	• Hyperendemic setting (Regional survey data) • Clinical grading: detailed WHO-FPC system, with more detailed grading of corneal scarring on photographs.[3] • MDA had been delivered in this region of Ethiopia several times before the start of the study and during the two year period. • One eye per person analysed. • 87/603 (14.5%) had a deterioration in visual acuity of >0.3 LogMAR units by 2 years. Most of this was not associated with a deterioration in CO, suggesting that other causes such as cataract were responsible.

## Results/Discussion

### Progression from Active to Scarring Trachoma

We identified four prospective cohort studies that reported the relationship between active trachoma and the subsequent development of incident scarring (new conjunctival scarring in those with no prior visible conjunctival scarring), Table 1 [10–13]. Three of these were from Tanzania and one from Tunisia. In addition, we identified a report of a mathematical model of the five year incidence of TS, based on cross-sectional age-stratified data from Tanzania [14]. The study designs were heterogeneous in terms of sample size, clinical grading system used, MDA treatment background and study duration. All four were conducted in hyperendemic communities, which had not previously received mass drug administration (MDA) for trachoma control. After enrolment all the communities received some form of antibiotic treatment, although this was variable in nature and frequency (Table 1). The duration of the follow-up varied from 5 to 14 years. The assessment of clinical signs used either the simplified or detailed WHO Trachoma Grading Systems [2, 3]. In two studies the development of incident scarring was identified by a change in the clinical field grading [10, 11]. In the other two studies the development of incident scarring was identified through the grading of photographs, using a more detailed scar grading system [12, 13].

The earliest study, from Tunisia, found that 17% of individuals (n = 82, children and young adults) without conjunctival scarring at baseline developed severe scarring with distortion of the tarsal plate (C3) over a 14 year period (Table 1) [10]. This study also reported on scarring progression and incident TT, which are discussed below. It did not report the proportion of individuals who developed milder degrees of scarring during the same period, nor did it report chlamydial infection data. A strong association was found between the presence of TI at baseline and the subsequent development of severe scarring (RR = 18), and a weaker association for the presence of TF (RR = 2.8). Unfortunately, the reported details are fairly limited, and the scarring incidence rates are not disaggregated by gender.

The first Tanzanian study was a 7-year cohort of children (baseline age 1–7 years) with and without “constant severe trachoma” (defined by the presence of TI on at least 3 out of 4 examinations during the baseline year) [11]. At 7 years, 29.2% of children with “constant severe trachoma” (n = 96) developed incident TS, compared to only 9.6% of the comparison group (n = 94). Incident scarring was independently associated with “constant severe trachoma”, increasing age and female gender. Incident scarring was associated with the presence of TI at the 7-year time-point. The cohort was tested for *C*. *trachomatis* infection at the 7-year time-point only; those that had incident scarring were more likely to be infected at that point (OR 2.48, p = 0.02).

In a separate cohort of Tanzanian children (n = 183) who were aged <10 years at baseline, the 5-year cumulative incidence of TS was 17.4%. In a logistic regression model, the development of TS was strongly associated with increasing age, female gender, “constant severe trachoma” (10+ follicles or TI or both, on at least 3 of 5 visits during the initial 18 months) and/or “constant *C*. *trachomatis* infection” (infection on at least 3 of 5 visits during the initial 18 months), compared to those without constant severe trachoma or constant infection, Table 1 [12]. The infection and disease states were not analysed as separate variables.

In the fourth prospective study to investigate incident conjunctival scarring in previously unaffected individuals, a cohort of Tanzanians of all ages (n = 367) was recruited and followed for 5 years (Table 1) [13]. The overall cumulative incidence of TS was 20.4% in 5 years. The cumulative incidence of scarring generally increased with age, although it was noted it was particularly rapid in younger children, for reasons that could not be determined. In this study the relationship between incident TS and the signs of active trachoma or the presence of infection were not reported. The study found that at baseline and at five years, across all age groups, the cross-sectional prevalence of TS was higher in females than males. However, data disaggregated by gender were not presented on the 5-year incidence of new scarring in this cohort.

The data from a mathematical model supports these longitudinal studies. The 5-year cumulative incidence of new scarring in females, based on cross-sectional data, indicated increasing incidence with increasing age: 3.1% in 15–19 year olds and 14.3% in 55–59 year olds [14].

Overall, there are surprisingly few prospective studies and relatively limited data investigating the link between active trachoma and/or chlamydial infection and the development of incident TS. Three studies present consistent evidence that severe conjunctival inflammation (TI) is associated with substantially increased risk of new scarring several years later [10–12]. The evidence from these studies for an association between TF, in the absence of TI, and the development of scarring is less clear. In the Tunisian study there was an increase in risk associated with TF (RR = 2.8), however, no test statistics (95%CI) were provided to assess the strength of this evidence [10]. In the Tanzanian populations TF was common and affected most of the children at some point in the observation periods [11, 12]. However, those that were found to have only sporadic disease or infection were not at a significantly higher risk of incident TS than those with no episodes of disease or infection [12].

The evidence from these prospective studies demonstrating a link between repeated or persistent *C*. *trachomatis* infection and subsequent incident TS is limited to only one study from Tanzania [12]. In this study, data on disease and infection were combined for the analysis, so it is not possible to determine the independence of the effect of infection from disease. Notably, although a small proportion of individuals with only sporadic disease and/or infection did develop TS, they were not at significantly greater risk of scarring than individuals who had no disease or infection episodes documented. This might suggest that in a programmatic context, reducing the pressure of infection and associated TI through MDA might reduce disease in the majority of individuals below a threshold required for the development of scarring.

As would be expected from the known cross-sectional age-specific prevalence of TS from population based surveys, the three prospective Tanzanian studies and the mathematical model found clear and consistent evidence of increasing risk of incident TS with increasing age [11–14]. However, it is noteworthy that some incident scarring was developing during childhood in these populations. Similarly, the prospective Tanzanian studies found that females were at consistently higher risk of incident TS than males, probably because of a greater lifetime exposure to chlamydial infection [11–13].

West and colleagues suggested that there might be two distinct routes to the development of TS [11]. Firstly, in the “classical” model, which may account for the majority of TS cases, are individuals who are repeatedly exposed to *C*. *trachomatis*. In this group the risk of TS is primarily determined by the cumulative number of infection episodes experienced. This is consistent with the observation that TS incidence is higher in females and with increasing age. This model has since been incorporated into the design of mathematical models of the scarring sequelae of trachoma [15, 16]. The second route, which may account for a minority of people who develop TS, is characterised by the development of a severe and persistent inflammatory phenotype, “constant severe trachoma”. Data from several of these prospective studies is consistent with this hypothesis [10–12]. Whether these individuals are responding unfavourably to *C*. *trachomatis*, to other bacteria, or are genetically prone to develop a severe persistent inflammatory phenotype is unclear [4].

### Progression of Scarring Trachoma

We identified four prospective cohort studies which reported the rates and risk factors for the progression of established trachomatous conjunctival scarring (Table 2) [10, 13, 17]. One was from Tunisia, one from Ethiopia and two from Tanzania. There was some heterogeneity in the study designs, although the two parallel studies we conducted in Ethiopia and Tanzania used similar designs and were reported together [17]. Three studies were conducted in hyperendemic communities [10, 13, 17] and one in a hypoendemic community [17]. The Tunisian and both Tanzanian studies were in areas which had not previously received MDA [10, 13, 17]. After enrolment, three of the study populations received some form of antibiotic treatment for trachoma control, although this was variable in nature and frequency (Table 2). The Ethiopian study was conducted in an area that had previously received MDA on several occasions and continued to do so during the study period [17]. The second Tanzanian study was conducted in an area where the current prevalence of active trachoma is below treatment thresholds [17]. The duration of follow-up varied from 2 to 14 years. The assessment of clinical signs used either the simplified or detailed WHO Trachoma Grading Systems [2, 3].

The identification of progressive scarring used different methodologies. In the Tunisian study the development of scarring was based on a change in the clinical field grading score [10]. In the other three studies the progression of scarring was identified through the grading of photographs. For one study this was done by the comparison of an independent grading of the baseline and five-year photographs [11]. In the other two studies the baseline and two-year photographs were graded using a detailed grading system for scarring [18]. The photographs were directly compared side-by-side for assessment of progression, possibly allowing for more subtle changes in TS to be detected [17].

The Tunisian study (described above) reported scarring progression in 68.5% of individuals (n = 120) over 14 years (Table 2) [10]. However, this report is limited in the level of detail provided and it appears that the total progression estimate may include some individuals who were incident scarring cases. There is some partial information on progression from mild or moderate scarring (C1 / C2) to the most severe grade (C3), Table 2. The specific risk factors for progression to C3 are not presented. However, as noted above, the development of new C3 in those who had no scarring at baseline was associated with TI at baseline.

In the first Tanzanian study progression of conjunctival scarring occurred in 47.1% of individuals (n = 85, all ages) who had some established TS at baseline, over a five year period (Table 2) [13]. There was no difference by age in the proportion with evidence of progression. No data on the relationship between scarring progression and gender, clinical inflammation or *C*. *trachomatis* infection were reported.

In our study in Ethiopia, progression of conjunctival scarring developed in 23.1% of participants (n = 585) over a 2 year period (Table 2) [17]. This was a prospective cohort of adults who had minor trichiasis at baseline that was managed by epilation. They were examined and photographed every six months, with swab samples collected for *C*. *trachomatis* PCR and conjunctival gene expression analysis. There was a strong relationship between progressive scarring and increasing number of observed episodes of clinical inflammation, defined as P2 or P3 in the detailed WHO grading system (OR 5.93, 95%CI 3.3–10.6, p<0.0001). There was no evidence of an association between scarring progression and gender, age or body mass index (BMI). The samples from individuals with progressive scarring and a frequency matched sample of individuals who did not show progressive scarring were tested for *C*. *trachomatis* by PCR on 4 occasions; all samples were negative for *C*. *trachomatis*. Conjunctival inflammation, but not scarring progression, was associated with increased gene expression of a mixture of pro-inflammatory cytokines (*IL1B*, *IL17A*, *CXCL5*), anti-microbial effectors (*S100A7*) and factors associated with tissue remodelling (*CTGF*, *MMP7*, reduced *SPARCL1*).

In the parallel cohort study we conducted in Tanzania we used the same protocol, including sample collection, as the Ethiopian study; all individuals had conjunctival scarring at baseline, were aged 18 years or more and no individuals had baseline trichiasis [17]. Progression of conjunctival scarring was observed in 30.0% of participants (n = 577) over a 2-year period (Table 2). Again, there was a strong relationship between progressive scarring and an increasing number of episodes of clinical inflammation (P2/P3), of a similar magnitude to that found in the Ethiopian study (OR 5.76, 95% CI 2.6–12.7, p<0.0001). There was no evidence of an association between scarring progression and gender, age or BMI. In this Tanzanian cohort there were 804 people with TS recruited and tested for *C*. *trachomatis* by PCR at baseline, of which only 4 samples were positive. At 6, 12 and 18 months after baseline, the samples from the individuals with progressive scarring and a frequency matched sample of individuals in the cohort who did not show progressive scarring were also tested for *C*. *trachomatis* and only one sample was positive. In this study progressive scarring was associated with a modest increase in expression of *IL1B* and S100A7. In addition, increased *S100A7*, *IL17A*, *CXCL5*, *MMP7* and *CEACAM5* expression were associated with clinical inflammation (P2/P3).

A consistent finding across these studies was that trachomatous conjunctival scarring continues to progress over time. The studies are probably too heterogeneous to provide an overall estimate range for disease progression. However, it is noticeable that in the two studies using the same protocol the rates of scarring progression were similar [17]. Moreover, it might have been anticipated that the Ethiopian cohort, which was conducted in a hyperendemic region, would have experienced a greater proportion of progression than that observed in a region of Tanzania that had a low level of active trachoma, and was therefore not included in the national antibiotic distribution programme.

There was a strong association between conjunctival inflammation and progressive scarring, with individuals experiencing more inflammatory episodes being at greater risk of progression [10, 17]. In two studies the immunofibrogenic correlates of clinically apparent inflammation were investigated using gene expression analysis, and this demonstrated that its presence was associated with increased expression of a range of pro-inflammatory factors [17]. These observations are consistent with the findings for incident scarring outlined above; children who experienced more episodes of active trachomatous inflammation were more likely to develop incident scarring (Table 1) [11, 12].

There are three noteworthy findings from these four cohort studies of progressive scarring. Firstly, we found no published evidence that the progression of established scarring was associated with the detection of *C*. *trachomatis* infection [17]. Indeed, infection was detected in only very few individuals with TS. This contrasts with the observation, albeit from limited prospective data, of an association between the development of incident scarring and episodes of chlamydial infection [12]. Secondly, we found no published evidence of a difference between males and females in the proportions showing signs of scarring progression. Again, this contrasts with the findings for incident scarring outlined above, which developed more frequently in females (Table 1). Thirdly, amongst people with established scarring the proportion showing scarring progression did not vary with age. This also contrasts with the findings for incident scarring, which developed more frequently with increasing age.

Therefore, once TS is established the risk of scarring progression does not appear to be associated with exactly the same set of risk factors that were associated with the development of incident scarring. This could be interpreted as suggesting that progression of established scarring is less dependent than incident scarring on recurrent *C*. *trachomatis* infection, episodes of which are more frequent in females and accrue with increasing age. It may also indicate that other factors such as non-chlamydial bacterial infection, a dry ocular surface or altered tissue inflammatory or scarring responses may also contribute to a pro-inflammatory / pro-fibrotic state. This might be anticipated as scarred ocular surface tissue probably has altered physiology and compromised defence mechanisms. However, prospective data on these factors has not been reported.

### Progression to Trachomatous Trichiasis

We identified four prospective cohort studies which report rates and risk factors for the development of trachomatous trichiasis (Table 3) [10, 19–21]. One was from Tunisia, one from Tanzania and two from The Gambia. In addition, we identified a mathematical model based on cross-sectional data from Tanzania [14]. Two studies were conducted in hyperendemic areas and two in hypoendemic areas. None of the study populations had previously received systematic MDA for trachoma control. After enrolment, two of the study populations received some form of antibiotic treatment for trachoma control, although this was variable in nature and frequency (Table 3) [10, 21]. The duration of follow-up varied from 5 to 14 years.

The Tunisian study (described above) reported incident TT in 8.0% over a 14 year period [10]. The risk of developing TT was related to the baseline conjunctival scarring severity, being 1.2% in people with no scarring and rising to 38.1% in those with severe baseline scarring (C3), Table 3. The Tanzanian study was a 7 year cohort of females with and without TS at baseline [19]. In those with TS at baseline 9.2% developed TT, and of those without TS at baseline 0.6% developed TT by 7-years. The development of TT was associated with increasing age and the detection of *C*. *trachomatis* infection at the 7-year time-point. The number of individuals with *C*. *trachomatis* infection at follow up was 60/513 (11.7%) in the group with TS at baseline and 36/501 (7.5%) in the group with no TS at baseline. The first of the Gambian studies reported the 12-year incidence of TT in people with TS at baseline to be 6.4% [20]. The development of TT was associated with increasing age. The second Gambian study was a five year study of all the residents of 14 villages, most (94%) of whom did not have scarring at baseline. The five year cumulative incidence of TT in those >15 years was 0.4% [21].

The mathematical model estimated the 5 year cumulative incidence of TT in women from a hyperendemic region of Tanzania using age-stratified cross sectional data from different clinical stages [14]. The cumulative incidence of TT was highest in older women (6.4% versus 0.3% in young women) and in women with TS at baseline (15.1% in 55–59 year olds, 3.2% in 15–19 year olds).

### Progression of Trachomatous Trichiasis

We identified three prospective cohort studies that reported the rates and risk factors for the progression of trachomatous trichiasis from unilateral to bilateral disease, or from minor trichiasis (<6 lashes touching the eye) to major trichiasis (6+ lashes touching the eye), Table 4 [22–25]. Two of these were from The Gambia and one from Ethiopia. The Gambian studies were both conducted in hypoendemic areas. The Ethiopian study was in a hyperendemic setting with on-going MDA. The duration of follow-up varied from 1 to 4 years.

In the first Gambian study, 46% of people with unilateral trichiasis (n = 46) had developed bilateral disease in one year [22]. Of those with minor trichiasis (n = 55), 33% progressed to major trichiasis. In the second Gambian study 29% of people with unilateral trichiasis (n = 42) progressed to bilateral disease in four years and 37% of minor trichiasis (n = 75) progressed to major trichiasis [23]. In this second study several factors were analysed in relation to the progression of trichiasis over four years: age, gender, conjunctival inflammation (at four years), chlamydial infection and pathological bacterial infection (at four years). In univariate analysis only conjunctival inflammation at 4-years was associated with TT progression. However, no factor was significant in a multivariable model. It is noteworthy that at 4-years both conjunctival inflammation and bacterial infection were independently associated with the presence of major trichiasis. Only 2/146 samples tested were positive for *C*. *trachomatis* at the 4-year time-point.

The Ethiopian study was of a group of people with minor trichiasis (n = 650) who were in the epilation arm of a randomised controlled trial of epilation vs. surgery, with regular follow up over a two year period [24]. Individuals were equipped with high quality forceps and a relative was trained in how to perform epilation. During the first two years 13% of study eyes progressed from minor to major trichiasis. After two years all participants were offered surgery for their trichiasis. About a third accepted surgery at two years, while the majority (n = 383) chose to continue with epilation and were followed up for an additional two years [25]. Comparing the amount of trichiasis by counting the absolute number of lashes touching the eye at baseline with that at four years, 21.4% of these 383 individuals were found to have had some degree of increase in the number of lashes touching the eye, however this was difficult to assess in the context of regular epilation. In addition, 36.6% had only a modest increase in the degree of entropion, although it should be noted that this is a variable and difficult clinical sign to grade. In a multivariable model, over the four years, progression to major trichiasis was associated with older age and having ≥3 lashes touching the eye at baseline.

### Progression to Corneal Opacification, Visual Impairment and Blindness

We identified four prospective cohort studies that reported the rates and risk factors for the development of corneal opacification (CO) and visual impairment (Table 5) [20, 22–25]. Three were from The Gambia, conducted in hypoendemic areas. One study was from a hyperendemic area of Ethiopia, with on-going MDA. The duration of follow-up varied from 1 to 12 years. In addition, the mathematical model based on Tanzanian cross-sectional data provided 10 year estimates for the development of CO [14]. We have not included prospective data on CO change from clinical trials where TT was treated with surgery.

The first Gambian study found the 12-year cumulative progression from TS to CO to be 5.9% and for TT to CO to be 20% [20]. Risk factors for incident CO were increasing age and TT at baseline. Visual impairment developed in 16.5%, but only 2.5% of this was attributed to CO; most was due to cataract (Table 5). The second Gambian study found that in one year incident CO developed in 10% of people with TT and that CO progressed in 34% with major trichiasis [22]. New visual impairment or blindness developed in 9%. The third study from The Gambia found incident CO developed in 7.6% of eyes with TT at baseline over the course of four years, with the risk being greater for those with major TT (Table 5) [23]. Again, the risk of visual impairment was increased with TT, however, the large majority of incident cases of visual impairment were due to cataract.

The four year Ethiopian study of people with minor trichiasis has been outlined above [24, 25]. Incident or progressive CO was determined by the comparison of photographs in this study. At 2 years, 5.5% of the 650 individuals with minor trichiasis had some increase in CO. When modelled at 4-years, incident/progressive CO was associated with being ≥50 years of age and having moderate CO at baseline. Visual acuity deteriorated in 14.5% by >0.3LogMAR by 2 years, however, most of this was not attributable to changes in CO.

The mathematical modelling study, described above, found that the overall incidence of CO was higher in older women: 2.8% in 45–54 years versus 0.16% in 15–24 years [14]. The cumulative incidence rates were estimated to be very much higher in those with TT at baseline: 53.5% in 45–54 year olds and 27.2% in 15–24 year olds. This model estimated around half of all CO was due to causes other than trachoma. In women <35 years these other causes dominated, whereas in older women trachoma was the main cause.

### Conclusions

Conducting long-term prospective studies of the rates and risk factors of progressive trachoma from active disease through to blindness is complex and expensive. Here we have reviewed the longitudinal studies that contribute to our understanding of this disease process. In addition to these longitudinal studies, there are many cross-sectional studies that provide indirect evidence about risk factors for progression. The prospective studies are relatively few in number, of variable design and frequently small in size. The determination of the presence of progression in conjunctival scarring can be challenging, particularly where there is also extensive inflammation masking scar tissue. Despite these limitations, collectively they provide evidence that supports much of the widely accepted description of the natural history of trachoma, illustrated in Fig 1.

The studies of incident and progressive conjunctival scarring were consistent in showing a strong association with repeated or persistent conjunctival inflammation. In contrast, the evidence linking the development of incident conjunctival scarring with *C*. *trachomatis* infection is limited to only one study. Moreover, in two large cohorts with scarring progression rates of 23.1% and 30% over 2 years only very few episodes of chlamydial infection were detected.

The model proposed by West and colleagues, and outlined above, suggests that the majority of individuals who develop TS do so after repeated rounds of infection by *C*. *trachomatis*. In addition, there appears to be a subgroup of individuals who experience repeated or protracted intense inflammation, who are at increased risk of developing scarring. It is anticipated that the introduction of control programmes involving MDA and F and E interventions to limit transmission, will reduce the number of episodes of infection experienced and therefore the risk of developing incident scarring. However, the potential impact of control measures on halting progression of previously established scarring is less certain. A treatment intervention that could reduce chronic conjunctival inflammation in adults in trachoma-endemic areas might be desirable to prevent scarring progression.

The lack of a clear association between chlamydial infection and scarring progression in adults could have a number of explanations [17]. It is possible that the number and frequency of clinical observations and tests for chlamydial infection in these studies were too few to capture infection events. It is also possible that the detection of infection in adults is more difficult than in children, because of lower infection loads or shorter infection duration, due to the acquisition of some protective immunity [26–28]. This potential explanation might imply that only brief bursts of infection with *C*. *trachomatis* is needed to trigger chronic inflammation and an associated scarring response.

Alternatively, it is possible that conjunctival inflammation observed in adults with established conjunctival scarring may have additional causes besides *C*. *trachomatis*. A number of studies have found cross-sectional associations between non-chlamydial bacteria cultured from the conjunctival surface and the presence of inflammation at all stages of the natural history of trachoma [23, 29–33]. Currently however there are no longitudinal data to determine whether this inflammation associated with other infections represents a pro-fibrotic state that contributes additional scarring, or whether it is a by-product of the altered physiological environment of a scarred conjunctiva. Longitudinal studies that include tests for *C*. *trachomatis* and non-chlamydial bacteria are needed to determine their relative contributions to scarring progression.

These observations raise important issues of programmatic significance for trachoma control, particularly over whether conjunctival scarring can continue to develop and progress in individuals with intermittent or chronic inflammation, but in the absence of on-going regular chlamydial infection. Several long-term population based studies of the impact of MDA have demonstrated that even after *C*. *trachomatis* has been cleared or brought down to very low prevalence levels, the prevalence of signs of active conjunctival inflammation persist for some years [21, 34]. While it is possible that the cross-sectional nature of these studies may have missed recent infection episodes, it is striking that the inflammatory phenotype was still present after the prevalence of *C*. *trachomatis* infection had been reduced to a low level, and this may have significance for disease progression. A question worth consideration is whether the clinical sign TI should be included in the monitoring of trachoma control in addition to TF, because of the consistently strong association between TI and incident and progressive scarring. Finally, these data support the recommendation that trachoma control programmes maintain mechanisms to detect and treat trichiasis for many years to come.
